# A Class of Rate-Independent Lower-Order Gradient Plasticity Theories: Implementation and Application to Disc Torsion Problem

**DOI:** 10.3390/ma11081425

**Published:** 2018-08-14

**Authors:** Emin Semih Perdahcıoğlu, Celal Soyarslan, Emin Erkan Aşık, Ton van den Boogaard, Swantje Bargmann

**Affiliations:** 1 Chair of Nonlinear Solid Mechanics, Faculty of Engineering Technology, University of Twente, 7500AE Enschede, The Netherlands; e.e.asik@utwente.nl (E.E.A.); a.h.vandenboogaard@utwente.nl (T.v.d.B.); 2 Chair of Solid Mechanics, School of Mechanical Engineering and Safety Engineering, University of Wuppertal, 42119 Wuppertal, Germany; soyarslan@uni-wuppertal.de (C.S.); bargmann@uni-wuppertal.de (S.B.)

**Keywords:** GND, SSD, strain gradient, crystal plasticity, size dependence

## Abstract

As the characteristic scale of products and production processes decreases, the plasticity phenomena observed start to deviate from those evidenced at the macroscale. The current research aims at investigating this gap using a lower-order gradient enhanced approach both using phenomenological continuum level as well as crystal plasticity models. In the phenomenological approach, a physically based hardening model relates the flow stress to the density of dislocations where it is assumed that the sources of immobile dislocations are both statistically stored (SSDs) as well as geometrically necessary dislocations (GNDs). In the crystal plasticity model, the evolution of the critical resolved shear stress is also defined based on the total number of dislocations. The GNDs are similarly incorporated in the hardening based on projecting the plastic strain gradients through the Burgers tensor on slip systems. A rate-independent formulation is considered that eliminates any artificial inhomogeneous hardening behavior due to numerical stabilization. The behavior of both models is compared in simulations focusing on the effect of structurally imposed gradients versus the inherent gradients arising in crystal plasticity simulations.

## 1. Introduction

In metals, the primary mechanism of inelastic deformation is plastic slip due to dislocation movement along glide planes. Due to the inhomogeneous nature of the microstructure, however, this movement is hindered by obstacles and, hence, prevails resistance to slip. According to [1] the magnitude of resistance depends on the number and type of obstacles. Since during deformation at low temperatures (with respect to the melting point of the metal) most microstructural features such as the grain morphology, number and composition of inclusions, the chemical composition of phases can be assumed to remain unaltered, the main mechanism that contributes to the change of resistance is the net change in the density of immobile dislocations causing dislocation pile-up.

During plastic slip, this number increases due to generation mechanisms such as Frank-Read sources and decreases due to dislocation annihilation where usually the net effect remains non-negative (at low temperatures where recovery and recrystallization are negligibly slow). These type of dislocations are commonly referred to as statistically stored dislocations (SSDs) due to the statistically random nature of their generation. This randomness results in a vanishing closed-circuit evaluation of the Burgers vector [2].

Another source of dislocations is geometrical necessity, as proposed by [3]. When large strain gradients prevail in the material an incompatibility of the crystal lattice arises which can be seen as a non-vanishing Burgers vector over a closed circuit. This implies that dislocations with certain orientations must be generated in order to reduce elastic distortion of the lattice. This type of dislocations is commonly referred to as geometrically necessary dislocations (GNDs).

Apart from their sources, however, both types of dislocations, locally, are in essence the same and, hence, contribute to the hardening of the material similarly in terms of creating dislocation pile-ups.

If the thermodynamics of the total deformation is considered, two types of state variables, namely the plastic slip and its gradient, contribute to the dissipation of free energy of the system. In other words, both terms can be considered as thermodynamic fluxes which must have associated thermodynamic forces. The force associated with slip is the Cauchy stress itself in the macroscopic approach and the critical resolved shear stress in the microscopic definition. For the gradient of slip, the associated thermodynamic force is the microscopic stress [4,5,6]. The presence of an additional dissipative mechanism demands further external forces to drive deformation. This corresponds to plastic hardening. For this type of models in which the plastic strain or the slip rates are defined as individual degrees of freedom, it is also necessary to define a constitutive law between the microscopic stress and the respective gradient term.

In the literature, based on their consideration of the microscopic stresses in the definition of the (micro)equilibrium, there are mainly two approaches regarding strain gradient plasticity. According to the terminology defined in [7], the models that do take this into account are referred to as higher-order and those who relate the enhanced hardening to GNDs only are referred to as lower-order approaches.

In the higher-order approach, in addition to microscopic stresses, additional boundary conditions related to the added degrees of freedom must be defined with respect to the weak-form of the boundary value problem. At the boundaries, which can also be the grain boundaries for the crystal plasticity framework, this gives more flexibility in defining realistic phenomena such as permeability of dislocations across the grain boundaries. On the other hand, inaccurate description of the physics at the boundaries of a structure may result in peculiar and even counterintuitive behavior [8].

In this work, we adopt a lower-order approach represented by two distinct plasticity models, namely macroscopic plasticity and crystal plasticity. In previous studies the effects of GNDs have been included in macroscopic plasticity models by considering an underlying phenomenological hardening function and relating the gradient of the strain either to the equivalent strain term or an additional stress multiplier through the use of a length-scale parameter [4,9,10]. On the other hand, it is possible to directly relate the GNDs to a hardening function that is based on dislocation densities to start with, such as the one proposed by Bergström [11], thereby circumventing the use of an arbitrary length scale parameter. Although this hardening function is originally developed considering the evolution of SSDs, in this study this formulation is enriched by introducing GNDs that evolve with the plastic strain gradients in order to determine the flow stress of the material.

In previous research on gradient enhanced crystal plasticity models mostly rate dependent theories have been introduced [12,13,14,15,16,17,18,19,20,21] with some exceptions such as [22,23,24,25,26]. In this study, a rate-independent crystal plasticity formulation is used, in which, slip only occurs on slip planes where the resolved shear stress value is larger than the slip resistance [27]. The hardening on each individual slip system is described by the total dislocation density. GND densities are computed by projecting the slip gradient on each system, on the slip vectors, further classified into edge or screw types. The evolution of SSDs are modeled based on observations of their generation mechanisms. The contribution of the total dislocation densities to the slip resistance on their respective as well as related slip systems are obtained using an interaction matrix. Finally, Taylor’s relation is utilized in relating the total dislocation densities to the critical resolved shear stress.

The computation of the gradients of slip and plastic strain is done using an explicit formulation after each converged time step in an implicit, non-linear FE simulation. The result is a weak coupling of the slip gradient and the equilibrium solution. Accordingly, within each increment a small error is introduced that is corrected in the following step.

The noteworthy features of the current work are:

1. A comparison of gradient enhanced theories with phenomenological and micromechanical plasticity models is carried out considering their formulations as well as their associated responses in disc torsion simulations.
2. As opposed to the commonly used rate-regularized formulations in the context, rate-independent plasticity models are considered. This choice is crucial especially for problems where strain rate gradients prevail for which viscous regularization might cause an uneven and non-physical treatment of material points in the whole problem domain.
3. The used discrete gradient method does not require a regular lattice. Moreover, the explicit nature of the utilized implementation does not impose a restriction on the selected finite element technology. Thus, the developed gradient framework proves to be robust and mesh independent.

The following notations are used: Consistently assuming a, b, and c as three second-order tensors, together with the Einstein’s summation convention on repeated indices, c=a·b represents the single contraction product with $c_{ik}=a_{ij}b_{jk}$. $d=a:b=a_{ij}b_{ij}$ represents the double contraction product, where *d* is a scalar. C=a⊗b represents tensor product with $C_{ijkl}=a_{ij}b_{kl}$. $a^{⊤}$ and $a^{-1}$ denote the transpose and the inverse of a, respectively. The gradient operator is defined using notation: ∇({•})=∂({•})/∂x.

## 2. Theory

In the following, a short summary of the underlying macroscopic and crystal plasticity constitutive models are given. The theory behind gradient enhancement that is included in the lower-order approach solely based on the additional hardening mechanism due to the generation of dislocations is given next in terms of the hardening effect and the relation of GND density and the strain gradients.

### 2.1. Constitutive Models

#### 2.1.1. Macroscopic Plasticity

We utilize a small strain theory in which the plastic and elastic strain rates additively make up the total strain rate that the material point encounters

(1) $$\dot{\varepsilon }={\dot{\varepsilon }}^{e}+{\dot{\varepsilon }}^{p}\;.$$

The elastic stress follows Hooke’s law

(2) $$\sigma =C^{e}:\varepsilon^{e}\;.$$

The plastic flow is taken as associative and, therefore, the normality rule applies

(3) $${\dot{\varepsilon }}^{p}=\dot{\gamma }\frac{\partial \phi }{\partial \sigma },$$

where $\dot{\gamma }$ is the plastic multiplier and ϕ is the yield function which is defined in the form

(4) $$\phi =\sigma^{\mathrm{eq}}-\sigma^{f}.$$

$\sigma^{\mathrm{eq}}$ is the equivalent stress defined with respect to the choice of the yield surface and $\sigma^{f}$ is the flow stress which is conventionally a function of only the equivalent plastic strain or its rate.

#### 2.1.2. Crystal Plasticity

Our point of departure is the additive decomposition of the displacement gradient H=∇u

(5) $$H=H^{e}+H^{p}\;.$$

Here, the elastic distortion, which describes the stretch and rotation of the underlying lattice, is given by $H^{e}$. $H^{p}$ denotes the plastic distortion which is associated with the cumulative slip activity of individual slip systems. Due to the history dependent nature of the plastic deformation, we continue with the rate form of this equation

(6) $$\dot{H}={\dot{H}}^{e}+{\dot{H}}^{p}\;.$$

Taking the symmetric part of above relation, one recovers the rate form of Equation (1), that is,

(7) $$\dot{\varepsilon }={\dot{\varepsilon }}^{e}+{\dot{\varepsilon }}^{p}\;,$$

and the elastic stress is defined in the same way as in continuum plasticity

(8) $$\sigma =C^{e}:\varepsilon^{e}\;.$$

Denoting $s^{\left(\alpha \right)}$ as the slip direction and $m^{\left(\alpha \right)}$ as the slip plane normal of the slip system α, the shear stress τ, acting on slip plane α, is calculated using

(9) $$\tau^{\left(\alpha \right)}=\sigma :\left[s^{\left(\alpha \right)}⊗m^{\left(\alpha \right)}\right]\;\mathrm{or}\;\tau^{\left(\alpha \right)}=s^{\left(\alpha \right)}\cdot \sigma \cdot m^{\left(\alpha \right)}\;.$$

Following, the flow criteria per slip system is introduced as

(10) $$\phi^{\left(\alpha \right)}=\tau^{\left(\alpha \right)}-\tau^{f\;(\alpha )}\;,$$

where $\tau^{f(\alpha )}$ is the slip system specific critical resolved shear stress. The plastic velocity gradient tensor reads

(11) $${\dot{H}}^{p}=\sum_{\alpha =1}^{m}{\dot{\gamma }}^{\left(\alpha \right)}s^{\left(\alpha \right)}⊗m^{\left(\alpha \right)}\;,$$

from which the plastic strain rate can be recovered with

(12) $${\dot{\varepsilon }}^{p}=\frac{1}{2}\sum_{\alpha =1}^{m}{\dot{\gamma }}^{\left(\alpha \right)}\left[s^{\left(\alpha \right)}⊗m^{\left(\alpha \right)}+m^{\left(\alpha \right)}⊗s^{\left(\alpha \right)}\right]\;.$$

Since we limit the scope to small displacements we consider irrotational crystal plasticity which implies that the rotation of the slip system vectors, $s^{\left(\alpha \right)}$ and $m^{\left(\alpha \right)}$, as a result of the deformation are considered small and therefore not taken into account in the formulations.

### 2.2. Hardening

#### 2.2.1. Macroscopic Plasticity

The core component of strain gradient theories is the additional slip resistance due to the increase in the total number of dislocations by the creation of GNDs. The dependence of slip resistance on the number of dislocations are generally described using Taylor’s shear flow model [1]

(13) $$\tau =c\mu b\sqrt{\rho }\;.$$

Here, μ is shear modulus, τ is the slip resistance, *b* the Burgers vector length and *c* is the constant Taylor factor. No distinction is made in this equation with respect to the generation mechanism of dislocations and SSDs as well as GNDs contribute to the slip resistance identically. Hence, the total number can be decomposed additively into the SSD and GND contributions.

By keeping the same definition for slip resistance in both macroscopic models and in crystal plasticity a more direct comparison between the gradient enhancement on hardening can be obtained. Hence, a hardening function based on Equation (13) is chosen in the description of the relation between the flow stress and plastic strain according to Bergström [11,28]

(14) $$\sigma^{f}=\sigma_{0}+c\mu b\sqrt{\rho }.$$

Here, $\sigma_{0}$ describes the initial resistance to slip described by the Peierls-Nabarro and lattice friction stresses [1]. The constant, *c*, in this context, is a material parameter that incorporates the Taylor factor as well as the relation between the shear stress and the equivalent stress. Furthermore, Bergström describes the evolution of SSDs with plastic strain as

(15) $$\frac{{\dot{\rho }}_{\mathrm{SSD}}}{{\dot{\varepsilon }}^{p,\mathrm{eq}}}=U\left(\rho_{\mathrm{SSD}}\right)-\Omega \left(\dot{\varepsilon },T\right)\rho_{\mathrm{SSD}},$$

where ${\dot{\varepsilon }}^{p,\mathrm{eq}}=\sqrt{\frac{2}{3}\;{\dot{\varepsilon }}^{p}:{\dot{\varepsilon }}^{p}}$ is the differential equivalent plastic strain, *U* is a function representing storage of dislocations (immobilization) and Ω represents dynamic recovery by remobilization and annihilation. Furthermore, *U* can be approximated as $U=U_{0}\sqrt{\rho_{\mathrm{SSD}}}$ using a material constant $U_{0}$ in order to account for the change in the mean-free path due to immobilization of dislocations.

#### 2.2.2. Crystal Plasticity

In defining a similar hardening function for crystal plasticity, it is essential to take into account the interaction of different slip systems with each other through self and latent hardening relations. Latent hardening is described by a matrix given as $Q^{\left(\alpha \beta \right)}$, which provides flexibility in introducing complex interactions. The number of independent parameters in this matrix can be reduced to 6, ($Q_{0}$ to $Q_{5}$), as described in [22]. Finally, the hardening in slip system α is determined as a function of all other slip systems in the same crystal ($\rho^{\left(\beta \right)}$) as

(16) $$\tau^{f\;(\alpha )}=\tau_{0}+c\mu b\sqrt{Q^{\left(\alpha \beta \right)}\rho^{\left(\beta \right)}}\;,$$

where $\tau^{f\;(\alpha )}$ is the flow stress on slip system α. It is implied that, both SSDs and GNDs contribute to the hardening identically, therefore, $\rho^{\left(\beta \right)}$ is considered as the direct sum of the two types of dislocation densities.

Next, we propose that the SSD density evolution is based on a phenomenological constitutive law as given in ([29], Chapter 8) and ([22], Chapter 3)

(17) $${\dot{\rho }}_{\mathrm{SSD}}^{\left(\alpha \right)}={\dot{\gamma }}^{\left(\alpha \right)}/\gamma^{\infty }\left[\rho_{\mathrm{SSD}}^{\infty }-\rho_{\mathrm{SSD}}^{\left(\alpha \right)}\right]\;.$$

Here, $\rho_{\mathrm{SSD}}^{\infty }$ denotes the saturation density of statistically stored dislocations and $\gamma^{\infty }$ determines the rate at which saturation occurs w.r.t. γ.

The ODE in (17), when solved, results in a saturating hardening curve similar to the function used for the continuum plasticity approach.

### 2.3. GND Density

#### 2.3.1. Crystal Plasticity

In order to define the relation between the gradient plastic strain and the GND density, it is essential to start with the definition of the Burgers tensor G [30] via

(18) $$G=\nabla \times H^{p}\;.$$

Here $H^{p}$ is the plastic part of the displacement gradient as defined in Equation (5). G can be interpreted as the deformation incompatibility direction vector for a given normal to a plane [2,31]. In this regard, G amounts to a Burgers tensor that is linked to edge and screw dislocations of geometrically necessary kind [32] viz.

(19) $$G=\sum_{\alpha }\rho_{\mathrm{GND},⊝}^{\left(\alpha \right)}l^{\left(\alpha \right)}⊗s^{\left(\alpha \right)}+\sum_{\alpha }\rho_{\mathrm{GND},⊙}^{\left(\alpha \right)}s^{\left(\alpha \right)}⊗s^{\left(\alpha \right)}\;,$$

with the line direction given as $l^{\left(\alpha \right)}=m^{\left(\alpha \right)}\times s^{\left(\alpha \right)}$ and

(20) $$\rho_{\mathrm{GND},⊝}^{\left(\alpha \right)}:=-s^{\left(\alpha \right)}\cdot \nabla \gamma^{\left(\alpha \right)}\;\mathrm{and}\;\rho_{\mathrm{GND},⊙}^{\left(\alpha \right)}:=l^{\left(\alpha \right)}\cdot \nabla \gamma^{\left(\alpha \right)}\;.$$

Here, $\rho_{\mathrm{GND},⊝}^{\left(\alpha \right)}$ denotes the geometrically necessary edge dislocation density through slip system α, and $\rho_{\mathrm{GND},⊙}^{\left(\alpha \right)}$ is the geometrically necessary screw dislocation along the same slip system. It is worth mentioning that the sign of the densities can also be negative and they have the dimension of one over length, that is $\left[\rho_{\mathrm{GND},⊝}^{\left(\alpha \right)}\right]=\left[\rho_{\mathrm{GND},⊙}^{\left(\alpha \right)}\right]=\left[1/L\right]$. This allows computation of total geometrically necessary dislocations viz.

(21) $$\rho_{\mathrm{GND}}^{\left(\alpha \right)}=\sqrt{{\left[\rho_{\mathrm{GND},⊝}^{\left(\alpha \right)}\right]}^{2}+{\left[\rho_{\mathrm{GND},⊙}^{\left(\alpha \right)}\right]}^{2}}\;.$$

The dislocation densities computed using the expressions above are measures of incompatibility and do not relate to a specific material property. In order to achieve this connection, the total GND density given in Equation (21) is divided by the length of the material specific Burgers vector *b* to yield the materials science version of the dislocation density [31].

In case of large deformations where rotations and stretches have a significant effect on the geometry, the gradient computation needs to be adjusted with the update of the configuration as treated in [33,34].

#### 2.3.2. Macroscopic Plasticity

For the macroscopic plasticity models, the average density of GNDs that contribute to the hardening of the material is relevant. The evolution GNDs is described by the formulation proposed and used by [1,3,4,10] by relating it to the curl of the plastic strain

(22) $$\chi =\nabla \times \varepsilon^{p}.$$

The similarity of (22) and (18) is apparent, however, the Burgers tensor takes the gradient of plastic displacements which is not necessarily symmetric since it also includes the rotational components.

In order to relate χ to the GND density, an equivalent scalar measure is defined which amounts to its second invariant [35]

(23) $$\chi =\sqrt{\chi :\chi }.$$

Although not directly comparable with Equation (21), this is also a measure of the incompatibility caused by the strain gradients and an equivalent measure in order to take into account the arbitrariness in the direction. On the other hand, according to [10,35], it is possible to algebraically dissect the curl operator and find an intermediate operator which is the gradient of the plastic strain. By finding invariants of this gradient tensor and considering the norm of the resulting curl operation the following relation has been proposed

(24) $$\rho_{\mathrm{GND}}=\frac{2\eta^{p}}{b}.$$

Here, $\eta^{p}$ is an equivalent measure of the plastic strain gradient evaluated using the deviatoric part of the third order plastic strain gradient tensor

(25) $$\eta^{p}=\sqrt{c_{1}\eta_{iik}^{\prime }\eta_{jjk}^{\prime }+c_{2}\eta_{ijk}^{\prime }\eta_{ijk}^{\prime }+c_{3}\eta_{ijk}^{\prime }\eta_{kji}^{\prime }}$$

with

(26) $$\eta_{ijk}^{\prime }=\eta_{ijk}-\frac{1}{4}\left[\delta_{ik}\eta_{jpp}+\delta_{jk}\eta_{ipp}\right]$$

and

(27) $$\eta_{ijk}=\varepsilon_{ik,j}^{p}+\varepsilon_{jk,i}^{p}-\varepsilon_{ij,k}^{p}.$$

Here, $c_{1}$, $c_{2}$ and $c_{3}$ are scaling factors used for correction based on the type of loading applied. For general metal plasticity which is assumed and modeled as isochoric deformation, i.e., $\varepsilon_{kk}^{p}=0$, the deviatoric $\eta^{\prime }$ equals the total one, η. Furthermore, based on the definitions above and the algebraic form resulting from (25) it can be observed that with a choice of $c_{1}=0$, $c_{2}=\frac{1}{4}$ and $c_{3}=-\frac{1}{4}$ Equation (23) is exactly reproduced.

On the other hand, it is clear from these expressions that in this form they are not applicable to the flow theory approach where history of deformation is taken into account. Therefore, instead of the total deformations, Equation (27) is written as a function of the rate of plastic strain to yield

(28) $${\dot{\rho }}_{\mathrm{GND}}=\frac{2{\dot{\eta }}^{p}}{b}.$$

## 3. Implementation

Both algorithms as presented here are implemented as user subroutines UMAT in the Abaqus software (version 6.12).

### 3.1. Rate-Independent Macroscopic Plasticity

The equations for macroscopic plasticity are solved using a Generalized Return Mapping Algorithm [36]. Within this algorithm, the selected constitutive relations can easily be substituted by alternative ones.

Based on the Kuhn-Tucker conditions

(29) $$\dot{\gamma }\geq 0,\;\phi \leq 0,\;\dot{\gamma }\phi =0\;,$$

two residual functions are defined, one being the tensorial sum of the elastic and plastic strains and the other is the current scalar value of the yield function

(30) $$\begin{array}{cc} R^{\varepsilon }\left(\sigma ,\gamma \right) & =\dot{\varepsilon }-{\dot{\varepsilon }}^{e}-{\dot{\varepsilon }}^{p}\\ R^{\phi }\left(\sigma ,\gamma \right) & =\sigma^{\mathrm{eq}}-\sigma^{f}. \end{array}$$

The nonlinear equation set defined in Equation (30) can be solved by any suitable algorithm such as the Newton-Raphson method provided that second derivative of the yield function with respect to stress can be determined.

### 3.2. Rate-Independent Crystal Plasticity

In the rate-independent crystal plasticity implementation, the stress is updated following the conventional approach of return-mapping algorithms. The Kuhn-Tucker conditions are defined per slip system α as

(31) $${\dot{\gamma }}^{\left(\alpha \right)}\geq 0,\;\phi^{\left(\alpha \right)}\leq 0,\;{\dot{\gamma }}^{\left(\alpha \right)}\phi^{\left(\alpha \right)}=0\;.$$

The incremental plastic multiplier on each slip system is obtained by a suitable solution scheme of the residual function [27]

(32) $$R^{\left(\alpha \right)}=\phi^{\left(\alpha \right)}=\sigma \left(\gamma \right):\left[s^{\left(\alpha \right)}⊗m^{\left(\alpha \right)}\right]-\tau^{f\;(\alpha )}\left(\gamma \right).$$

In the implementation of rate-independent crystal plasticity, two issues arise: non-uniqueness and active set determination. The algorithms presented in [27,29,37] provide robust and efficient solutions to these issues.

Non-uniqueness occurs when at least two slip systems have exactly the same resolved shear stress and their mutual influence on their hardening is the same, such as in isotopic hardening. However, using singular value decomposition or the more efficient perturbation methods, the correct slip amounts are recovered. The active set α is determined by the condition $\phi^{\left(\alpha \right)}>0$. The issue that arises during iterations is that the active set might change as slip systems enter or leave the set. As long as the correct systems that activate and deactivate can be found, the solution is converged. Deactivation of the most violated system ($\Delta \gamma^{\left(\alpha \right)}<0$) is based on the condition

(33) $$\sum \phi^{\left(\alpha \right)}\Delta \gamma^{\left(\alpha \right)}<0\;.$$

### 3.3. Gradient Computation

In this work, computation of strain gradients is realized explicitly making use of a discrete gradient computation method proposed by Liszka and Orkisz [38], see also, [39,40]. This method allows computation of gradients for arbitrary irregular grids. To this end, the following first order Taylor series expansion of ${\dot{\gamma }}^{\left(\alpha \right)}$ around point $r_{0}=\left\{x_{0},y_{0},z_{0}\right\}$ is developed

(34) $$\begin{array}{c} {\dot{\gamma }}^{\left(\alpha \right)}\left(r\right)={\dot{\gamma }}^{\left(\alpha \right)}\left(r_{0}\right)+Y\cdot \Delta r+O\left(\Delta x^{2}+\Delta y^{2}+\Delta z^{2}\right)\;, \end{array}$$

computed for α=1,2,3,…,N where Y represents the unknown gradient vector

(35) $$Y={\left\{\partial {\dot{\gamma }}^{\left(\alpha \right)}\left(r_{0}\right)/\partial x,\partial {\dot{\gamma }}^{\left(\alpha \right)}\left(r_{0}\right)/\partial y,\partial {\dot{\gamma }}^{\left(\alpha \right)}\left(r_{0}\right)/\partial z\right\}}^{⊤}\;,$$

and Δr is defined as $\Delta r={\left\{\Delta x,\Delta y,\Delta z\right\}}^{⊤}$ with $\Delta x=x-x_{0}$, $\Delta y=y-y_{0}$ and $\Delta z=z-z_{0}$. In macroscopic plasticity, the scalar field that is evaluated becomes components of the plastic strain rate tensor.

Equation (34) is based on the assumption that ${\dot{\gamma }}^{\left(\alpha \right)}\left(r\right)$ is a sufficiently smooth field that can be computed at point r={x,y,z}. Writing Equation (34) for each Gauß point around the central Gauß point gives a set of linear equations. In order to overcome the over-determinacy of this system as well as providing a smooth approximation to the gradient, the following form is used

(36) $$f\left(Y\right)=\sum_{k=1}^{n}{\left[\frac{{\dot{\gamma }}^{\left(\alpha \right)}\left(r_{0}\right)-{\dot{\gamma }}^{\left(\alpha \right)}\left(r_{k}\right)+Y\cdot \Delta r_{k}}{\Delta_{k}^{3}}\right]}^{2}\;,$$

where $1/\Delta_{k}^{3}$ is the weighting factor. The desired gradients are found by minimizing f(Y) using ∂f/∂Y=0. The USDFLD subroutine found in Abaqus software is used for implementing this algorithm.

Other studies can be found where the gradients are computed using the FE interpolation functions within an element [41]. An advantage of the presented approach is that due to the least square fitting, the resulting gradients are smooth and less sensitive to numerical noise and jumps across element boundaries.

In the case of polycrystal simulations, the gradient computation is limited within each domain of elements belonging to individual material definitions. This implies that the jump of the plastic strain across the grain boundaries is not treated as a source of GNDs. In the literature, studies can be found describing grain boundary effects on GNDs such as [42,43,44].

### 3.4. Global Solution Scheme

In the weakly coupled scheme that is presented above, the gradient effects do not directly influence the current equilibrium solution increment. On the other hand, the introduced GNDs (as a result of the increase in the gradients of plastic deformation) affect the hardening response of the material. This additional hardening term only influences the next increment in the equilibrium solver, making the current algorithm a staggered one.

The general equilibrium solution scheme in the commercial finite element software Abaqus with emphasis on the utilized user-defined subroutines reads:

**Algorithm 1.** Equilibrium solution scheme including gradient computation and user defined material in the commercial FE software package Abaqus.⋮ **increment**
*n* USDFLD: assign $\nabla {\dot{\varepsilon }}^{p}$ or $\nabla {\dot{\gamma }}^{\left(\alpha \right)}$
(α=1,2,…,N) → SDV UMAT: global iterations 1,2,…,k using SDV USDFLD: compute $\nabla {\dot{\varepsilon }}^{p}$ or $\nabla {\dot{\gamma }}^{\left(\alpha \right)}$
(α=1,2,…,N) **increment**
n+1 USDFLD: assign $\nabla {\dot{\varepsilon }}^{p}$ or $\nabla {\dot{\gamma }}^{\left(\alpha \right)}$
(α=1,2,…,N) → SDV UMAT: global iterations 1,2,…,k using SDV USDFLD: compute $\nabla {\dot{\varepsilon }}^{p}$ or $\nabla {\dot{\gamma }}^{\left(\alpha \right)}$
(α=1,2,…,N) ⋮

## 4. Application and Results

### 4.1. Disc Torsion

**Figure 1:**
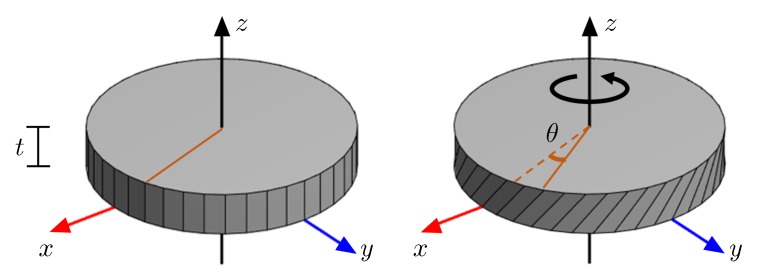
Considered disc during finite element simulations. Making use of cylindrical coordinates (r,ψ,z) with x=rcosψ, y=rcosψ, during loading bottom surface with z=0 is fixed completely with $u_{r}\left(r,\psi ,0\right)=u_{\psi }\left(r,\psi ,0\right)=u_{z}\left(r,\psi ,0\right)=0$ whereas at the top surface with z=t, torsional loading along z-axis is applied with $u_{r}\left(r,\psi ,t\right)=0$, $u_{\psi }\left(r,\psi ,t\right)=\theta$ and $u_{z}\left(r,\psi ,t\right)=0$. The shear strain, $\varepsilon_{\psi z}$, is kept constant for different radii of discs by scaling the thickness with the radius as: t=0.24r.

The geometry and boundary conditions considered in this test are depicted in Figure 1. Accordingly, in this test, the top and bottom sides of the disc are constrained and a twist (θ) is applied at the top. Wire torsion tests [4] suggest that as the disc radius decreases the additional hardening mechanism due to the applied strain gradient should become more dominant; resulting in a difference in the measured normalized torque. The torque (τ) is calculated as the reaction force to the applied twist to the upper surface.

The shear component that is the main deformation mechanism is $\varepsilon_{\psi z}$ (see Figure 1) and it varies linearly in the radial direction when the deformations are prescribed on both surfaces, i.e., $\varepsilon_{\psi z}=0.5\;r\;\theta /t$. The crucial distinction relating to this problem in the two presented approaches in the theory section is the fact that the macroscopic approach takes a structural gradient as a source of GNDs whereas GNDs form naturally in the crystal plasticity approach. Even if there is no structural gradient, the existence of grains with distinct lattice orientations creates strain gradients close to the grain boundaries. This results in an inherent hardening mechanism.

**Figure 2:**
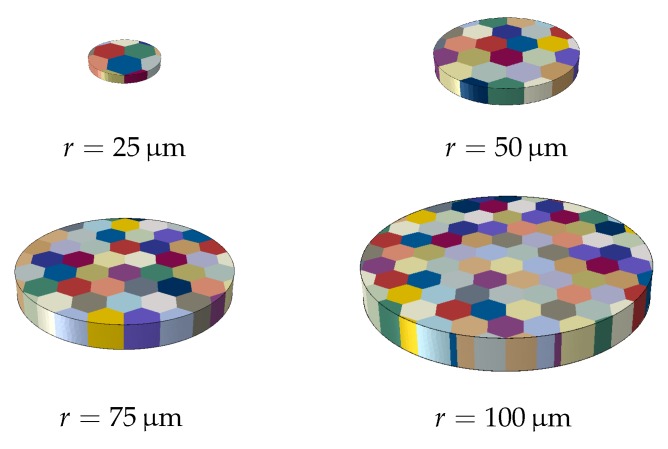
Microstructure of the discs used in the crystal plasticity simulations. Every crystal (represented by a different color) has a different crystallographic orientation.

As the disc diameter decreases, the grain size of the material remains constant if not explicitly altered. Due to the nature of the wire-drawing process, through which the disc is assumed to be cut, it is expected that the grains in the disc are elongated in the primary axis of the wire. Therefore, the simulation models shown in Figure 2 are created in order to mimic the experiments as closely as possible. The approximate grain diameter is chosen to be 25μm.

The structure is meshed using quadratic tetrahedral elements with 4 integration points. The same mesh sizes are used for crystal plasticity and macroscopic plasticity, which is chosen to yield 72 elements per full-size grain.

### 4.2. Parameter Identification

Since the aim of the simulation is to observe and compare the gradient enhanced hardening behavior of the two models, the underlying plastic hardening response should be made as similar as possible. On the other hand, the crystal plasticity model has an inherent dependence on the strain gradient which arises due to the existence of grain boundaries even in the absence of an imposed structural gradient. Hence, the macroscopic model is fitted to the crystal plasticity results in the case of a test with no structural strain gradient, which is chosen to be a plane-strain tension test. The grain size is set identical to that in the torsion simulations.

**Figure 3:**
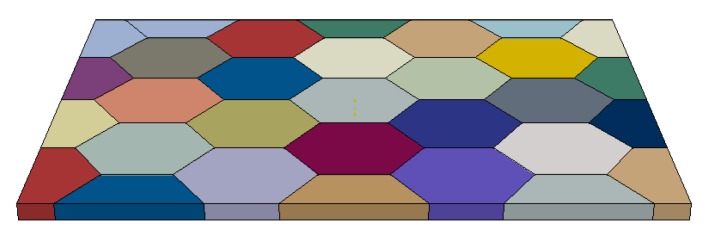
3D polycrystal crystal plasticity simulation including gradient effects where each color represents a different grain orientation.

**Figure 4:**
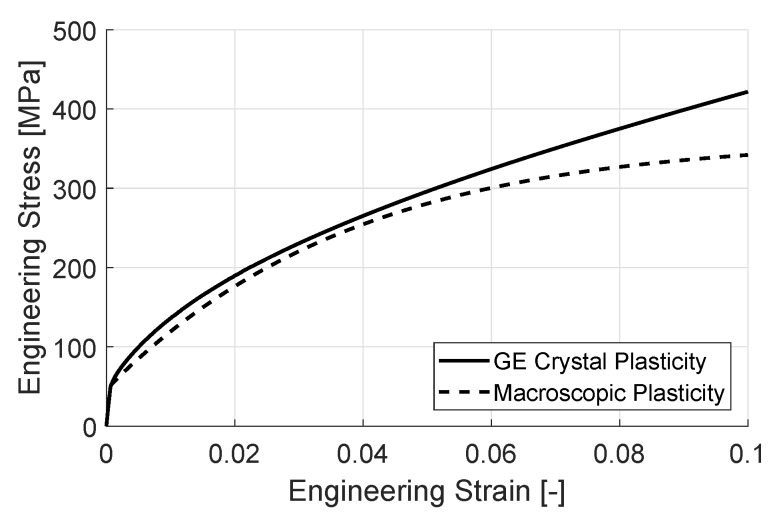
Numerical plane strain tension test. Fitted hardening curve of the macroscopic plasticity model to a 3D polycrystal crystal plasticity simulation including gradient effects.

The simulations are carried out in 3D in order to achieve a realistic fit - due to the orientation of the slip systems out-of-plane components of strain gradients contribute to the hardening of the material. An *fcc* crystal structure is chosen and the complete set of slip systems are utilized in the computation. The material data, which is based on an Aluminum alloy, used for both cases are summarized in Table 1 and the model and the fit are shown in Figure 3 and Figure 4, respectively.

The two models, macroscopic and crystal plasticity, have a good agreement at the starting stages of hardening, see Figure 4. As the strain increases, the macroscopic model starts to saturate in terms of flow stress, whereas the gradient enhanced crystal plasticity model does not and continues to harden with an almost constant slope. The saturation of the macroscopic model is expected due to the dislocation density evolution law, i.e., Equation (15). Although the crystal plasticity hardening behavior is chosen also as saturating, the creation of GNDs depend on the strain gradients and are, therefore, independent of the evolution of SSDs. This non-saturating behavior is also observed in experimental studies found in literature for Aluminum as well as Steel [28,45].

### 4.3. Results and Discussion

**Figure 5:**
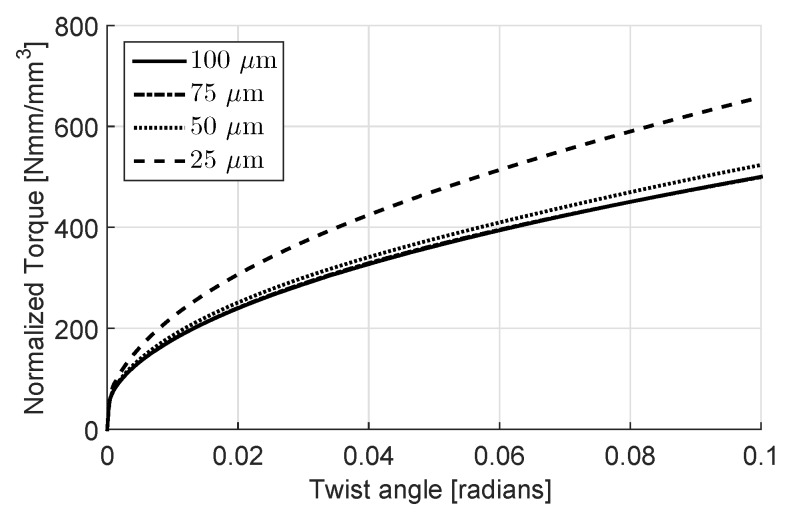
Torque vs. twist angle curves with varying disc radii.

The resulting normalized torque ($\tau /r^{3}$) versus twist angle curves for the crystal plasticity case are plotted in Figure 5. Although there is only a minor amount of hardening down to a disc radius of 50 μm, below that a significant change occurs.

**Figure 6:**
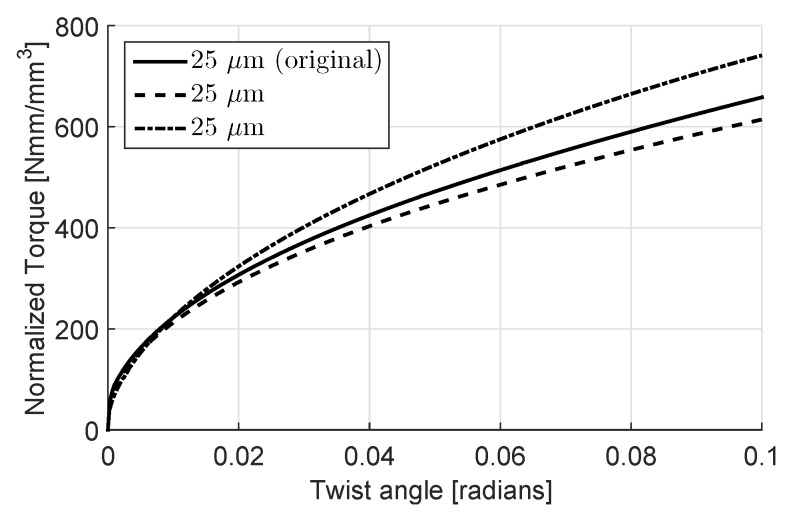
Torque vs. twist angle curves with 25μm disc radius and 2 sets of additional randomly generated orientations.

**Figure 7:**
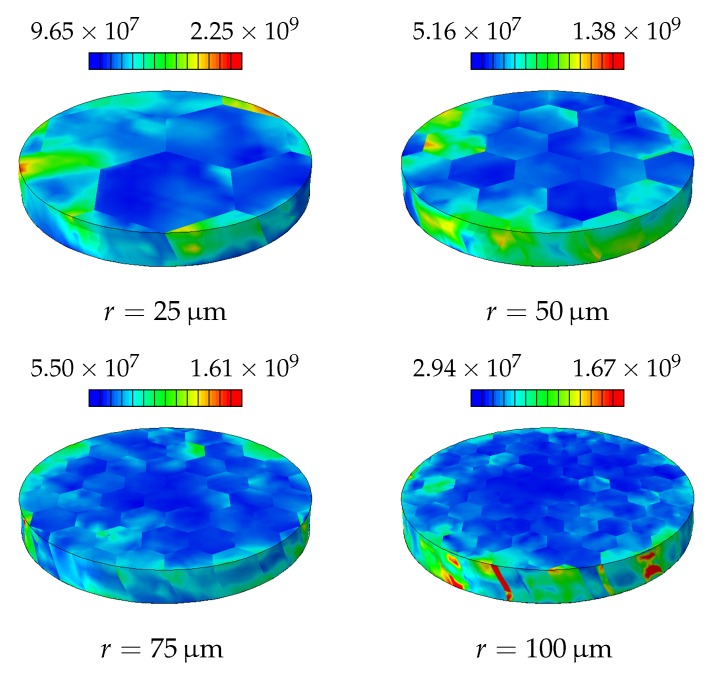
Final distribution of GNDs $\left[{\mathrm{mm}}^{-2}\right]$ in the disc as a result of the applied torsion.

**Figure 8:**
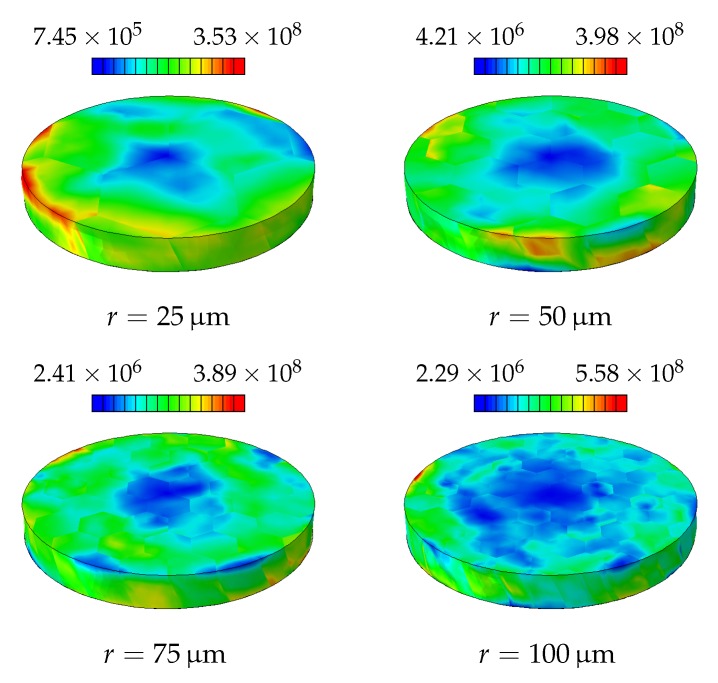
Final distribution of SSDs $\left[{\mathrm{mm}}^{-2}\right]$ in the disc as a result of the applied torsion.

One reason for this is as expected the increased strain gradient due to the boundary conditions. On the other hand, at these diameters only a few grains, hence crystallographic orientations, are available for generating slip, see Figure 2. Therefore, this simulation is repeated twice with randomly generated crystallographic orientations in order to observe the statistical nature of the hardening. The statistical size effect, as a result of highly constrained plastic deformation, becomes a significant mechanism for hardening as the disc radius decreases, see Figure 6. The resulting GND and SSD distributions for different disc diameters are shown in Figure 7 and Figure 8, respectively.

**Figure 9:**
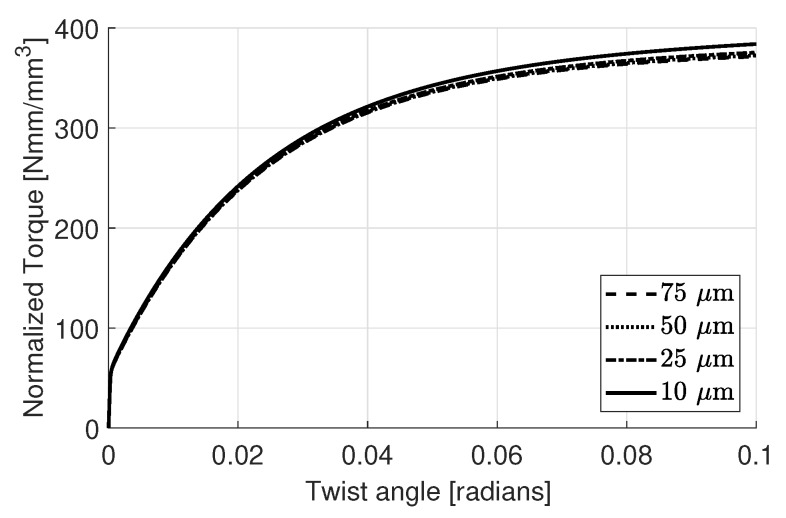
Torque vs. twist angle curves with varying disc radii using macroscopic plasticity approach.

The normalized torque and twist angle curves for simulations of the disc using gradient enhanced macroscopic plasticity are shown in Figure 9. At a first glance the results seem to imply that the hardening effect due to GNDs are underestimated by the macroscopic approach. On the other hand, as discussed before, the crystal plasticity simulations also show a minor effect down to 50μm which corresponds well with these observations considering the fitting of the material parameters in that the same order of magnitude is captured. As also mentioned before, it is believed that below this scale the statistical hardening effect becomes the dominant mechanism which cannot be captured with a macroscopic model.

**Figure 10:**
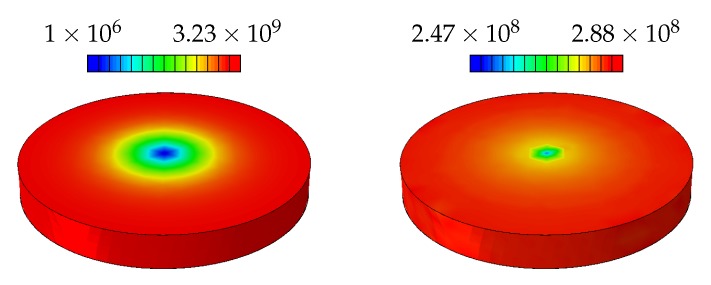
Distribution of the final SSDs (**left**) and GNDs (**right**) $\left[{\mathrm{mm}}^{-2}\right]$ for the disc with r=10μm, as a result of the applied deformation.

A representative case (radius = 10μm) for the SSD and GND distributions for the macroscopic plasticity case are shown in Figure 10. Here as expected the distributions are more homogeneous since the only source of GNDs is the structural strain gradient. The SSD distribution shows a saturation towards the outer edge due to the nature of the Bergstrom hardening in which the SSD generation and annihilation reach a steady state which causes the hardening to saturate at high strains. At the center of the disc there is a small elastic region where no plasticity occurs. Since the GND formation only occurs in the plastic regime at this point a lower value is observed due to the interpolation of the fields at the integration points.

It should be noted that since the main deformation mechanism in the torsion simulation is shear, care needs to be taken with respect to the effects of rigid rotation. In the simulations above, dominantly, small strains can be assumed. The phenomena that is observed start to occur already at the small strains and therefore cannot be attributed to either rigid or crystal rotations.

## 5. Conclusions

Gradient enhanced plasticity theories stem from the micromechanical observations that when the gradient of strain in crystalline materials reaches certain levels, the number of GNDs that need to be generated in order to accommodate the incompatibility of deformation reaches similar orders of magnitudes as the SSDs. The source of the gradient can be structural, due to strain concentrations or imposed gradients, or microstructural due to inhomogeneities such as obstacles, second phases and more importantly grain boundaries. In order to assess the two approaches that exist in the literature an explicit gradient computation algorithm is added to a commercial FE program which can utilize the underlying arbitrary integration point distribution for the computation. It is shown that it works successfully in both cases for macroscopic as well as crystal plasticity models.

Two approaches are implemented which stem from the same underlying theory. Due to its nature the macroscopic model only captures structural gradients. The same type of hardening is used for both models for valid comparison based on dislocation densities.

GNDs naturally appear at the grain boundaries and, therefore, contribute to the overall hardening behavior of the material regardless of the existence of a structural gradient. The macroscopic hardening model is consequently fitted to a simulation that includes this effect.

Both models are able to predict hardening due to this structural gradient, but the hardening effect seems small. On the other hand, below a certain size another size dependent mechanism starts to dominate the hardening behavior. Due to the limited number of slip systems existing in the disc, the plasticity is much more constrained. Therefore, depending on the crystallographic orientations, a harder response is observed. This is tested with different generations of orientations and it is shown that, statistically, the change in hardening is much more significant compared to the effect of the structural gradient.

## Figures and Tables

**Table 1:** Material data used in simulations.

Continuum Plasticity				Crystal Plasticity		
*E*	[MPa]	75,000		*E*	[MPa]	75,000
μ	[-]	0.3		μ	[-]	0.3
*b*	[mm]	$2.86\times 10^{-7}$		*b*	[mm]	$2.86\times 10^{-7}$
c	[-]	0.57		c	[-]	0.3
$\rho_{0}$	[mm${}^{-2}$]	$3\times 10^{5}$		$\rho_{0}$	[mm${}^{-2}$]	$2\times 10^{5}$
*U*	[mm${}^{-1}$]	$2.85\times 10^{6}$		$\rho^{\infty }$	[mm${}^{-2}$]	$3\times 10^{8}$
Ω	[-]	50		$\gamma^{\infty }$	[-]	0.75
$\sigma_{0}$	[MPa]	45		$\tau_{0}$	[MPa]	10
				$Q_{0-5}$	[-]	2,10,10,10,8,15
